# Prevalence and Risk Factors for *Bartonella* spp. and Haemoplasma Infections in Cats from Greece

**DOI:** 10.3390/vetsci9070337

**Published:** 2022-07-03

**Authors:** Kassiopi Christina G. Kokkinaki, Manolis N. Saridomichelakis, Vassilis Skampardonis, Antonia Mataragka, John Ikonomopoulos, Leonidas Leontides, Mathios E. Mylonakis, Joerg M. Steiner, Jan S. Suchodolski, Panagiotis G. Xenoulis

**Affiliations:** 1 Clinic of Medicine, Faculty of Veterinary Science, University of Thessaly, 224 Trikalon Str., GR-43132 Karditsa, Greece; msarido@vet.uth.gr (M.N.S.); pxenoulis@vet.uth.gr (P.G.X.); 2 Laboratory of Epidemiology, Biostatistics and Economics of Animal Production, Faculty of Veterinary Science, University of Thessaly, 224 Trikalon Str., GR-43132 Karditsa, Greece; bskamp@vet.uth.gr; 3 Laboratory of Anatomy and Physiology of Farm Animals, Department of Animal Science, Agricultural University of Athens, 75 Iera Odos, Votanikos, GR-11855 Athens, Greece; stud13856@aua.gr (A.M.); ikonomop@aua.gr (J.I.); 4 Companion Animal Clinic, School of Veterinary Medicine, Aristotle University of Thessaloniki, 11 Stavrou Voutyra Str., GR-54627 Thessaloniki, Greece; mmylonak@vet.auth.gr; 5 Gastrointestinal Laboratory, Department of Small Animal Clinical Sciences, College of Veterinary Medicine and Biomedical Sciences, Texas A & M University, College Station, TX 77843, USA; jsteiner@cvm.tamu.edu (J.M.S.); jsuchodolski@cvm.tamu.edu (J.S.S.)

**Keywords:** feline, molecular, prevalence, risk factors, serology, vector-borne

## Abstract

**Simple Summary:**

Bartonellosis and haemoplasmosis are diseases with global impact on the health of domestic cats and of zoonotic importance. This is the first study investigating the risk factors for *Bartonella* spp. and haemoplasma species infections in cats from Greece. In addition, this study determined the serologic and molecular prevalence of *Bartonella* spp. and haemoplasma species infections in different populations of cats living in different regions of Greece. A total of 452 cats were enrolled into the study. Blood was collected from each cat for the serological detection of *Bartonella henselae* antibodies and the molecular detection of *Bartonella* spp. and haemoplasma species infections. Overall, the seroprevalence of *B. henselae* was 35.4%, while the molecular prevalence of *Bartonella* spp. and haemoplasma species was 2.9% and 19%, respectively. The results of this study indicate that cats with partial or exclusive outdoor access and cats with flea infestation are at the greatest risk for *B. henselae* seropositivity. Furthermore, cats living in warmer regions such as Attica and Crete are at the greatest risk. Lack of ectoparasiticide use was identified as a risk factor for haemoplasma species infection. This suggests that the use of ectoparasiticides in cats may be an effective means of preventing haemoplasma species infection in cats.

**Abstract:**

Bartonellosis and haemoplasmosis are vector-borne diseases with global impact on the health of domestic cats and of zoonotic importance. The aim of this study was to describe the epidemiological aspects of various populations of cats infected with *Bartonella* spp. or haemoplasma species. The populations evaluated included client-owned cats, stray cats and cats that live in breeding catteries in Greece. A total of 452 cats were prospectively enrolled into the study. A commercially available indirect immunofluorescence antibody testkit was used for the detection of Bartonella henselae IgG antibodies in serum. PCRs for the detection of Bartonella spp. and haemoplasma species DNA in the blood were also performed in a subgroup of 242 of the 452 cats. Risk factors for B. henselae seropositivity and infection with the haemoplasma species were determined using multivariable analysis. Overall, 160 (35.4%) of the 452 cats were seropositive for *B. henselae*. Seven (2.9%) and 46 (19%) of the 242 cats were PCR-positive for *Bartonella* spp. and haemoplasma species, respectively. The factors associated with *B. henselae* seropositivity, based on multivariate analysis, included older age, outdoor access, living region and flea infestation. Non-administration of ectoparasiticides was associated with haemoplasma species infection. This study shows a high prevalence of seropositivity for *B. henselae* and a relatively high prevalence of infection with haemoplasma species. Therefore, it is necessary to establish optimal strategies for the prevention of *Bartonella* spp. and haemoplasma species infections, considering the high-risk groups of cats identified in this study.

## 1. Introduction

Bartonellosis and haemoplasmosis are vector-borne diseases with global impact on the health of domestic cats and of zoonotic importance.

*Bartonella* species are small, Gram-negative intracellular bacteria and cats are the main reservoir of *Bartonella henselae*, *Bartonella clarridgeiae* and *Bartonella koehlerae* and accidental hosts for *Bartonella quintana*, *Bartonella bovis* and *Bartonella vinsonii* subsp. *berkhoffii*. The most common species infecting cats is *B. henselae*, which is the main causative agent of cat-scratch disease in humans [1]. The major mode of transmission among cats is by exposure to flea bites (*Ctenocephalides felis)* or faeces, and uncommonly by aggressive interactions among cats [2,3,4].

The reported serological and molecular prevalence of *Bartonella* spp. varies considerably, ranging between 0–85% and 0–83.5% [5], respectively, depending on the geographical location and study population. The most well-documented risk factor for *Bartonella* spp. infection is flea infestation [6]. In addition, younger cats, especially those under 2 years of age, are more likely to present with bacteraemia compared to older cats, while the latter appear to be more likely to become seropositive compared to the former [6,7,8,9,10,11,12]. Cats with outdoor access are more likely to become infected due to increased access to flea-infested environments [11,13,14,15,16,17,18]. Similarly, multi-cat households are a risk factor for *Bartonella* infection due to the increased number of potential hosts (other infected cats) and vectors [19].

Haemoplasmas are haemotropic mycoplasmas that infect erythrocytes. The main haemoplasma species currently known to infect cats are *Mycoplasma haemofelis*, *Candidatus Mycoplasma haemominutum* and *Candidatus Mycoplasma turicensis.* Although the natural mode of transmission of haemoplasma species among cats is not clear, arthropod vectors including *C. felis* and aggressive interactions with infected cats have been suggested [20].

The reported molecular prevalence of haemoplasma species infection ranges between 4% and 43% [21]. In several studies, male gender has been reported as a risk factor for haemoplasma species infection [20,22,23]. From a behavioural perspective, between-cat aggression is higher in male cats than in females, leading to a greater risk of bite wounds and haemoplasma transmission. Outdoor access and cat bite abscesses have also been suggested as risk factors [22]. Cats living outdoors or having outdoor access have increased chances of aggressive interactions with haemoplasma-infected cats and exposure to fleas. Non-pedigree cats are more likely to become infected compared to purebred cats [24]. The results of several studies indicate that older cats are more likely to be infected compared to younger cats [22,25]. A possible explanation for this association could be that older cats have more chance of acquiring chronic subclinical infections during their lives. As already mentioned, *C. felis* has been implicated in the transmission of feline haemoplasmas, despite the limited evidence for transmission by fleas under experimental or natural conditions [26]. However, the regular use of ectoparasiticides has been associated with a decreased risk of haemoplasma infection [27].

Only limited data are available on the epidemiology of *Bartonella* spp. and haemoplasma species in cats from Greece, with a total of four reported studies for both infections [28,29,30,31]. The risk factors for these two vector-borne pathogens were not investigated in any of these studies. In addition, these studies included cats living in certain parts of the country and admitted to veterinary clinics for veterinary care, likely representing a somewhat biased population that does not reflect the general feline population.

The objectives of the present study were to determine the seroprevalence of *B. henselae* and the molecular prevalence of *Bartonella* spp. and haemoplasma species in different populations of cats living in different regions of Greece, and to assess the risk factors for *B. henselae* seropositivity and haemoplasma species PCR positivity. Based on previously reported data, we hypothesized that the major risk factor for *B. henselae* seropositivity and haemoplasma species PCR positivity would be flea infestation.

## 2. Materials and Methods

### 2.1. Ethics Approval

The study protocol was reviewed and approved by the Animal Ethics Committee of the Faculty of Veterinary Science, University of Thessaly (13/16-6-15). Handling of these animals was in compliance with the European Communities Council Directive 2010/63/EU and state laws.

### 2.2. Study Population

**Figure 1:**
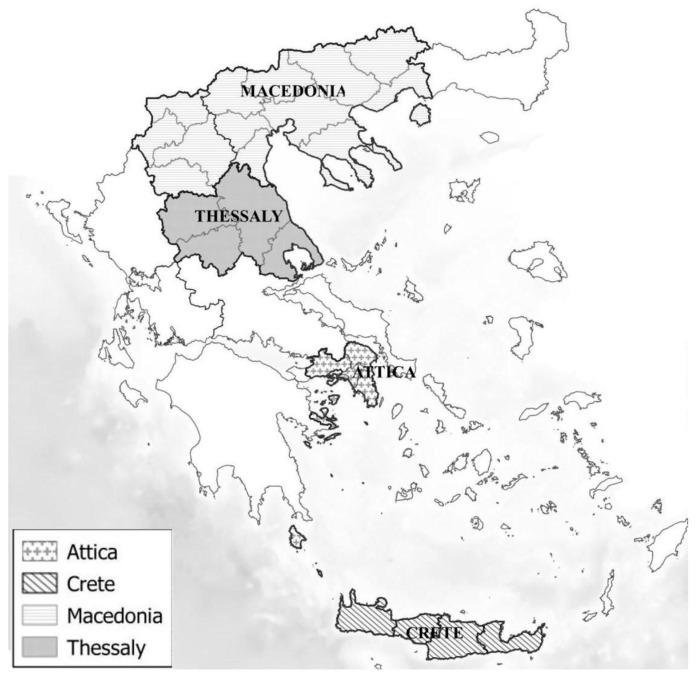
Map of the study area. Cats from the region of Attica, Crete, Macedonia and Thessaly were prospectively enrolled.

Cats from four different geographic areas of Greece (i.e., Attica, Thessaly, Crete and Macedonia) (Figure 1) were prospectively enrolled by three clinicians between November 2013 and November 2016 and divided into three groups: client-owned cats, stray cats and cats living in breeding catteries. A subgroup of these cats was described in a recently published study investigating the prevalence of FeLV antigenemia and FIV seropositivity, as well as the clinical signs and the haematological and biochemical findings associated with Feline leukemia virus and feline immunodeficiency virus infection [32]. The cats were presented by their owners (client-owned or breeding cattery cats) or by two cat-rescue groups (stray cats) for wellness examination, vaccination, neutering and/or medical treatment. Stray cats often originated from cat-dense environments (i.e., rescue colonies) and their age was estimated based on body size, dentition and other physical characteristics.

The inclusion criteria for the cats in all groups included: (a) a body weight of ≥0.5 kg; and (b) an informed consent form signed by the owner or the rescuer. Cats were considered healthy based on a normal physical examination and no recent history of disease. Cats who presented with systemic clinical (anorexia, depression, lethargy), gastrointestinal (vomiting, diarrhoea), ocular, neurological or dermatological signs (skin lesions and/or pruritus) were considered sick.

For the serological detection of *B. henselae* IgG antibodies, cats that fulfilled the above two criteria were enrolled on a sequential basis, regardless of their health status. For the molecular detection of *Bartonella* spp. and haemoplasma species, the cats were randomly selected from the initially enrolled cats using a randomisation table.

Signalment and historical data were collected using a standardised questionnaire (Questionnaire S1) and a thorough physical examination was performed for each cat by one of three authors (KGK, PGX, MEM). Historical information included: a) signalment; (b) geographic origin and travel history; (c) prior and current ownership; (d) living conditions (indoors, outdoors); (e) diet (dry food, canned food, home cooked food, raw meat); (f) vaccination status (fully vaccinated, partially vaccinated, unvaccinated); (g) parasite prevention status (regular use of ectoparasiticides, intermittent use, non-use); (h) prior medical problems; (i) previous treatments; (j) chief presenting complaint; (k) present health status (e.g., weight loss, reduced appetite, faecal characteristics).

The minimum required study population size to detect at least two times higher odds of *B. henselae* seropositivity among flea infested than non-infested cats, with 80% power at the 95% confidence level, was determined to be 418 cats (StatCalc, EpiInfo ver. 7.2.5; Centers for Disease Control and Prevention (CDC), Atlanta, Georgia).

### 2.3. Sample Collection and Laboratory Analyses

A total of 5 mL of blood was collected by jugular venipuncture from adult cats and 3.5 mL from young kittens. One millilitre of blood was sequentially transferred into two EDTA-anticoagulated tubes. One sample was used for haematology, while the other was stored at −80 °C for PCR. The remaining blood was transferred in an anticoagulant-free tube, and the serum was harvested following centrifugation at 3000 rpm for 20 min. The serum aliquots were stored at −80 °C until biochemical analysis and indirect fluorescent antibody test (IFAT) were conducted.

Haematology was performed using one of three different haematology analysers (Sysmex pocH-100i, Norderstedt, Germany; ADVIA 2120i Siemens Healthcare GmbH, Erlangen, Germany; ADVIA 120 Siemens Healthcare GmbH, Erlangen, Germany), depending on the geographic area of the sampling, and the results were classified as normal, increased or decreased, based on the reference intervals of each laboratory. For most cats, the differential white blood cell count was calculated manually, and the inspection for platelet aggregates was carried out in Diff-Quik (Merck, Darmstadt, Germany)-stained blood smears, whereas for the remaining cats (*n* = 25), it was based on the results of analysis using the ADVIA 2120i analyser, Siemens Healthcare GmbH, Erlangen, Germany. All haematologic analyses were performed within 12 h of blood collection. Biochemical analyses were performed using an automated chemistry analyser (Roche/Hitachi MODULAR ANALYTICS D 2400 module, Roche Diagnostics, Rotkreuz, Switzerland).

### 2.4. Serologic Testing

*Bartonella henselae* IgG antibodies were detected using commercially available IFAT kits (Biopronix Product Line, Agrlolabo S.p.a., Torino, Italy) according to the manufacturer’s instructions as previously described [33,34]. For each assay run, negative and positive controls that contained the IFAT kit were included. The cut-off value was 1/64 for *B. henselae*. All slides were examined by fluorescence microscopy at 40× magnification (Olympus, Tokyo, Japan).

### 2.5. Molecular Analyses

DNA was extracted from whole blood using the Nucleospin Tissue Kit (Macherey-Nagel GmbH & Co. KG, Düren, Germany), based on the instructions provided by the manufacturer, and stored at −20 °C until use.

The assessment of the DNA’s purity and integrity was conducted with submerged gel electrophoresis and image analysis using the Bio-Rad ChemiDoc XRS+ Molecular Imager (Bio-Rad Laboratories Inc., Des Plaines, IL USA). The DNA solutions were submitted for analysis of optical density count at 260/280 nm, using a NanoDrop 8000 Spectrophotometer (Thermo Fisher Scientific Inc., Watham, MA, USA). DNA solutions for which this part of the pre-analytical assessment provided evidence of fragmentation or low quality were discarded, and DNA isolation was repeated from the original sample. Samples with acceptable DNA quality were processed for the assessment of the presence of PCR inhibitors using a PCR assay targeting actin as a housekeeping gene [35]. DNA solutions that were negative for the housekeeping gene were diluted in ddH_2_O (1:5 *v*/*v*) and the analysis was repeated. Depending on the outcome, the diluted DNA solutions were either discarded and DNA isolation was repeated from the original sample, or processed for the detection of the study pathogens.

The DNA isolation and PCRs were performed using positive and negative controls integrated into each stage of the analysis (DNA/PCR positive and negative controls). With regard to DNA isolation, the control samples consisted of 500 μL ddH_2_O (DNA C-), and whole blood of equal volume (500 μL) that was PCR-positive for the respective pathogen, confirmed by sequence analysis of the PCR product (DNA C+). For the PCR, the positive and negative controls consisted of DNA solutions that were confirmed PCR-positive for each assay (PCR C+) or 5 μL of the DNA elution buffer (PCR C-), respectively.

PCR was performed in an Applied Biosystems Verity 96-well Thermal Cycler (Thermo Fisher Scientific Inc., Watham, MA, USA). Real-time PCR was performed in a Roche LightCycler 2.0 (Roche, Germany). The PCR products were analysed by submerged electrophoresis using 2% agarose gels stained with ethidium bromide (0.5 μg/mL) and visualised using a Bio-Rad ChemiDoc XRS+ Molecular Imager (Bio-Rad Laboratories Inc., Des Plaines, IL, USA). For confirmation of the specificity of the amplification products, approximately 20% of the PCR products were submitted for sequence analysis, which was conducted on both strands using the Applied Biosystems BigDye Terminator Cycle Sequencing Kit and PRISM 377 DNA Sequencer (Thermo Fisher Scientific Inc., Watham, MA, USA). The results were analysed and compared to deposited sequences in the GenBank database (http://www.ncbi.nlm.nih.gov; accessed on 12 January 2018) using the Basic Local Alignment Search Tool (BLAST) of the National Center for Biotechnology Information (NCBI).

The validation of the PCR assays used in our study was based on the ad hoc laboratory analysis of previously published methods. This analysis included the assessment of the method specificity using in silico analysis of the relevant oligonucleotide primers and limit of detection (LoD), as previously described [36]. The LoD was assessed on serial dilutions of DNA isolated from whole-blood samples that were PCR-positive to the respective assays, confirmed by the sequence analysis of the PCR products. The analysis was conducted in line with the provisions of the quality standard ISO17025:2017 (ISO/IEC 17025 (2017). General requirements for the competence of testing and calibration laboratories. International Organization for Standardization/International Electrotechnical Committee, Geneva).

### 2.6. PCR Protocol for Detection of Bartonella *spp.*

The target sequence was a 209 base-pair fragment of the *Bartonella ssrA* gene (Forward: GCTATGGTAATAAATGGACAATGAAATAA, Reverse: GACGTGCTTCCGCATAGTTGTC) [28]. A 25 μL mixture for each PCR reaction was performed with the Kapa Taq PCR kit following the standard protocol suggested by the manufacturer. The amplification profile consisted of an initial denaturation step at 95 °C for 5 min, followed by 30 cycles of incubation at 95 °C for 30 sec, 60 °C for 45 sec and a final elongation step at 72 °C for 5 min.

### 2.7. PCR Protocol for Detection of Haemoplasma Species

The target sequence was a fragment of the haemoplasma 16*S*-*rRNA* gene (Forward: ACGAAAGTCTGATGGAGCAATA, Reverse: ACGCCCAATAAATCCGRATAAT) [37]. A 25 μL mixture for each PCR reaction was performed with the Kapa Taq PCR kit, following the standard protocol suggested by the manufacturer. A hot-start PCR was performed, starting with 5 min at 95 °C. The amplification profile consisted of an initial denaturation step at 95 °C for 5 min followed by 30 cycles of incubation at 95 °C for 30 sec, 60 °C for 45 sec and a final elongation step at 72 °C for 5 min.

### 2.8. Statistical Analysis

The analyses were only performed on cats with complete information on all variables considered. Cats with missing data were excluded. In the univariate analyses, the categorical data regarding signalment and historical information in *B*. *henselae* seropositive cats or haemoplasma species PCR-positive cats were compared to those of *B*. *henselae* seronegative cats or haemoplasma species PCR-negative cats, respectively, by either Pearson’s χ^2^ or Fisher’s exact test. The normality of the distribution of the continuous variables was evaluated with the Kolmogorov–Smirnov test. Therefore, normally distributed data are presented as means ± standard deviation and were compared between PCR-positive or seropositive and PCR-negative or seronegative cats using two-sample t-tests. Variables whose distribution deviate from normality are presented as medians and ranges and were compared between PCR-positive or seropositive and PCR-negative or seronegative cats using Mann–Whitney *U* tests.

Subsequently, variables relating to signalment and historical information that were different at *p* < 0.25 between *B*. *henselae*-seropositive cats or haemoplasma species PCR-positive and *B. henselae*-seronegative or haemoplasma species PCR-negative cats, respectively, in the univariate analyses were selected as candidates for initial logistic regression modelling. The initial models were subsequently reduced in a stepwise manner until only the variables significant at *p* < 0.05 remained. Odds ratios (OR) derived from the reduced models were interpreted as measures of increased risk of seropositivity or PCR positivity. The analyses were conducted using Stata 13 (Stata Corp, College Station, TX, USA) and SPSS 23 for Windows (IBM Corp, Armonk, NY, USA).

## 3. Results

### 3.1. Serological Testing for B. henselae (n = 452)

A total of 452 cats were enrolled. The signalment and historical data are presented in Table 1. Of the 452 cats, 160 (35.4%; 95%CI 31–39.8%) were found to be seropositive for *B. henselae*. The univariate associations between seropositivity for *B. henselae* and signalment-historical data are presented in Table 2.

### 3.2. Risk Factors for B. henselae Seropositivity

Several factors were found to be significantly associated with *B. henselae* seropositivity in the multivariate analysis (Table 3). Seropositive cats were significantly older than seronegative cats; moreover, cats living outdoors were significantly more likely to be seropositive than cats living indoors. In addition, cats living in Attica and Crete were significantly more likely to be seropositive than cats living in Thessaly. Flea infestation was also found to be risk factor for *B. henselae* seropositivity.

### 3.3. PCR Validation

PCR validation showed that the PCR assays do not lead to false-positive results [28,37,38]. The analytical sensitivity of the PCR assay designed for the detection of DNA belonging to *B. quintana, B. henselae, B. bovis* and *B. elizabethae* was determined at 5 fg of DNA/reaction [38]. The relevant information is not available in connection with haemoplasma species, because the conditions for its in vitro isolation and accurate enumeration have not been described [37]. With regards to the in silico analysis of the oligonucleotide primers used for PCR assays, the specificity was found to be 100% based on sequence comparison using the Basic Local Alignment Search Tool (Primer-BLAST) [39] of the National Center for Biotechnology Information (NCBI). At the 98% level of confidence, the LoD determined ad hoc for the study PCR assays was 0.01 and 0.02 ng/mL for *Bartonella* spp. and haemoplasma species, respectively.

### 3.4. PCR Testing for Bartonella *spp.* and Haemoplasma Species (n = 242)

A total of 242 cats were selected for the molecular detection of *Bartonella* spp. and haemoplasma species infections. The signalment and historical data are presented in Table 1.

Of the 242 cats, a total of 7 (2.9%; 95%CI 0.7–5%) and 46 (19%; 95%CI 14.1–23.9%) were found to be PCR-positive for *Bartonella* spp. and haemoplasma species, respectively. Of the PCR-positive cats, three (1.2% of the total 242 cats; 95%CI 0.2–2.6%) had concurrent PCR-positive results for *Bartonella* spp. and haemoplasma species. The univariate associations between haemoplasma species and signalment/historical data are presented in Table 4.

### 3.5. Risk Factors for Haemoplasma Species PCR Positivity

The multivariate analysis indicated that cats who had not been treated with a preventative ectoparasiticide were significantly more likely to be haemoplasma species PCR-positive than cats who had been treated with ectoparasiticides (Table 3).

## 4. Discussion

This is one of the largest prospective studies on natural infection with *Bartonella* spp. and haemoplasma species in cats from Greece. In addition, this is the first study investigating the risk factors for these two vector-borne pathogens in cats from Greece. Overall, 35.4% of the cats were seropositive for *B. henselae*, while 2.9% and 19% of the cats were PCR-positive for *Bartonella* spp. and haemoplasma species, respectively. Older age, outdoor access, living region and flea infestation were significant predictors of *B. henselae* seropositivity. Non-administration of ectoparasiticides was associated with PCR positivity for haemoplasma species.

The haemoplasma species prevalence identified here by PCR is similar to the results from a previous prospective study in cats from Greece, where the prevalence was reported to be 20.6% [29]. However, in another study from Greece that assessed *Leishmania infantum* infection in 50 clinically healthy cats and 50 sick cats, the authors reported a seroprevalence of *B. henselae* of only 4% [31]. The higher prevalence in our study could be explained by differences in the study population. In the previous study, cats were enrolled from two certain areas of Greece (central and northern Greece) and included cats examined at veterinary clinics or enrolled in spay/neuter programmes. In our study, we enrolled a large number of cats of all ages, lifestyles and health status. In parallel with this, we aimed to have a study population representative, as much as possible, of the general feline population in Greece. Therefore, we included cats living both in the continental mainland and insular regions of Greece; regarding the former, we included cats living in the north (Thessaloniki), in the central (Thessaly), and in the south mainland (Attica) and regarding the latter, we included cats living on the largest island of Greece (Crete). Furthermore, the sites selected were based on the presence of colonies of stray cats and on the willingness of animal welfare organisations and local veterinarians to collaborate with us. More recently, in a study investigating the occurrence of zoonotic parasites and vector-borne pathogens in stray cats living in selected areas of Greece, the authors reported a seroprevalence of *B. henselae* of 58.8% [30]. Furthermore, in a retrospective study of 100 cats from Greece, 8.5% and 14.9% were PCR-positive for *Bartonella* spp. and haemoplasma species, respectively [28]. The seroprevalence for *Bartonella* spp. in cats has been reported to be 0–68% in Europe [40], 0–85.2% in the USA [10], 37% in Australia [41] and 11–59% in Africa [42]. Studies have reported an overall PCR-based prevalence of feline infection with haemoplasma species of 32.1% in South Africa [43], 27.2% in Australia [44], 26.4% in Asia [45], 12–27% in the USA [46] and 4–43% in Europe [47]. The variability of serologic and molecular prevalence among these studies may reflect differences in the demographics and the geographic origin of the cats, in the accuracy of the diagnostic tests, the presence of illness and/or anaemia and/or the overtime changes in the prevalence of these infections. For example, contact with other cats and outdoor access have been shown to increase the risk of *Bartonella* spp. infection. Furthermore, studies where cats were sampled from lower income areas have shown a high prevalence of vector-borne diseases compared to cats living in higher income areas, because such cats may be less likely to receive preventive ectoparasiticide treatment. Furthermore, different real-time PCR assays have been developed for *Bartonella* detection and have been used in studies. They have differences in their sensitivity, probes used and range of species detected.

In the current study, *B. henselae*-seropositive cats were significantly older than *B. henselae*-seronegative cats. This is consistent with previous studies suggesting that older cats are more commonly infected with *B. henselae*, possibly due to the fact that they have more time to be exposed to the microorganism [6,18].

Our study showed that cats with outdoor access were more likely to be *B. henselae*-seropositive compared to cats living strictly indoors and this is in agreement with previous reports, likely reflecting increased access to flea-infested environments [6,14].

In addition, cats living in Attica and Crete were significantly more likely to be *B. henselae*-seropositive than cats living in Thessaly. Although the reason for this finding is not known, it may be due to the slightly different environmental conditions in Thessaly compared to Attica and Crete. Attica and Crete have slightly higher average temperatures (about 2–3 degrees) almost all months of the year. *Ctenocephalides felis*, the most important arthropod vector for the transmission of *Bartonella* spp. in cats, is strongly influenced by environmental factors including temperature and humidity, and these environmental conditions may be more favourable in Attica and Crete than in Thessaly. Cats infested with fleas were more likely to be *B. henselae*-seropositive compared to cats without flea infestation. Many studies have documented that *B. henselae* is naturally transmitted among cats by the flea *C. felis* or by flea faeces [2], while *C. felis* was found to be the major flea species affecting cats in Greece [48]. In a recent study from Greece involving the molecular detection of *Bartonella* spp. in fleas and ticks parasitising cats and dogs from Attica, the authors demonstrated the presence of *B. henselae* and *B. clarridgeiae* in *C. felis* fleas from cats and dogs in Greece [49]. Our results indicating flea infestation as a risk factor for *B. henselae* seropositivity is in accordance with the results of previous studies [14,16,19]. In a recent study on zoonotic vector-borne pathogens in cats from Italy, a risk analysis revealed that cats with flea infestation were 3.5 times more likely to be *B. henselae*-seropositive than cats without flea infestation [6].

Cats who had not been on a preventative ectoparasiticide were more likely to be PCR-positive for haemoplasmas than cats previously treated with an ectoparasiticide. The mode of natural transmission of haemoplasma species in cats is still elusive, but fleas and other arthropod vectors are suspected to be involved. The cat flea *C. felis* has been implicated in feline haemoplasma transmission, but this has not been definitively proven experimentally. In an experimental study conducted in 2005 [50], a very transient *M. haemofelis* infection was reported via the haematophagous activity of fleas, but clinical and haematological changes consistent with *M. haemofelis* infection were not observed in the recipient cat. Furthermore, ticks have been proposed as potential vectors, since feline haemoplasma species have been found in ticks collected from cats [51]; however, no clear evidence for their role in the natural transmission of feline haemoplasma species exists. Similarly, mosquitoes have been proposed as vectors for feline haemoplasma species, but there is no evidence of the biologic transmission of feline haemoplasma species.

This study has some limitations. Although the cats enrolled were randomly selected, selection bias might still exist. Only cats that were examined at veterinary clinics and had an informed consent form signed by their owner or the rescuer were included. Owners willing to participate in research are more aware of the risks of vector-borne infections and the implementation of strict flea control measures, so they are more likely to use ectoparasiticides on their animals. Therefore, cats being on a preventative ectoparasiticide may have been overrepresented in our study. Another limitation of our study is that the diagnostic sensitivity and specificity of the IFAT kit used for the detection of *B. henselae* IgG antibodies were unknown. In addition, the lack of identification of *Bartonella* spp. and haemoplasmas on a species level in this study prevented the potential expansion of the spectrum of these pathogens affecting felines in Greece.

## 5. Conclusions

This study showed a high prevalence of seropositivity for *B. henselae* and a relatively high prevalence of haemoplasma species infection. The results of this study indicate that cats with partial or exclusive outdoor access and cats with flea infestation are at higher risk for *B. henselae* seropositivity. Furthermore, cats living in warmer regions such as Attica and Crete are at higher risk for *B. henselae* seropositivity. The lack of ectoparasiticide use was identified as a risk factor for haemoplasma species PCR positivity. This suggests that the use of ectoparasiticides in cats may be an effective mean of preventing haemoplasma species infection in cats.

Future prospective studies in naturally infected cats are warranted in order to investigate the clinical and clinicopathologic relevance of different species of *Bartonella* and haemoplasmas in cats in Greece and other geographic locations.

## Figures and Tables

**Table 1:** Signalment and historical data of 452 cats selected for serology detection of *B. henselae* infection and of 242 cats selected for molecular detection of *Bartonella* spp. and haemoplasma species infections.

		Serology		PCR	
Signalment/Historical Data	Categories	Number of Cats (%)	Missing Data (%)	Number of Cats (%)	Missing Data (%)
Age (years, range, median)		0.125–17 (2)	23 (5.1)	0.125–15 (1)	9 (3.7)
Sex	Male	241 (53.3)	0 (0)	130 (53.7)	0 (0)
	Female	211 (46.7)		112 (46.3)	
Neutered		138 (30.5)	6 (1.3)	71 (29.3)	3 (1.2)
Breed	Purebred	15 (3.3)	15 (3.3)	2 (0.8)	6 (2.5)
	Common European	422 (93.4)		234 (96.7)	
Living conditions	Indoors	97 (21.5)	22 (4.9)	30 (12.4)	7 (2.9)
	Outdoors (exclusively or partially)	333 (73.7)		205 (84.7)	
Geographic regions	Attica	255 (56.4)	0 (0)	94 (38.8)	0 (0)
	Crete	79 (17.5)		70 (28.9)	
	Macedonia	45 (10)		18 (7.4)	
	Thessaly	73 (16.2)		60 (24.8)	
Current ownership	Client-owned	267 (59.1)	9 (2)	137 (56.6)	2 (0.8)
	Stray	155 (34.3)		91 (37.6)	
	Cattery	21 (4.6)		12 (5)	
Living area	Urban	361 (79.9)	23 (5.1)	176 (72.7)	8 (3.3)
	Rural	68 (15)		58 (24)	
Health status	Clinically healthy	167 (36.9%)	11 (2.4)	74 (30.6)	3 (1.2)
	Sick	274 (60.6)		165 (68.2)	

**Table 2:** Univariable associations between *Bartonella henselae* seropositivity and the signalment and historical data that were collected using a standardised questionnaire (Questionnaire S1).

			*Bartonella henselae* IgG Antibodies		
Variables	Categories	Missing Data	Seropositive (%)	Seronegative (%)	*p* Value
Sex	Male	0	78/160 (48.8%)	163/292 (55.8%)	0.15 ^a^
	Female		82/160 (51.2%)	129/292 (44.2%)	
Neutered		6	49/157 (31.2%)	89/289 (30.8%)	0.928
Breed	Purebred	15	2/154 (1.3%)	13/283 (4.6%)	0.071 ^a^
	Common European breed		152/154 (98.7%)	270/283 (95.4%)	
Age (years)		23	2 (0.13–17)	1 (0.13–15)	<0.001 ^a^
Cat acquisition	Stray	93	87/118 (73.7%)	170/241 (70.5%)	0.529
	Non-stray		31/118 (26.3%)	71/241 (29.5%)	
Current ownership	Client-owned	9	94/157 (59.9%)	173/286 (60.5%)	0.081 ^a^
	Stray		60/157 (38.2%)	95/286 (33.2%)	
	Cattery		3/157 (1.9%)	18/286 (6.3%)	
Living conditions	Indoors	22	21/150 (14%)	76/280 (27.1%)	0.002 ^a^
	Outdoors		129/150 (86%)	204/280 (72.9%)	
Living area	Urban	23	120/149 (80.5%)	241/280 (86.1%)	0.135 ^a^
	Rural		29/149 (19.5%)	39/280 (13.9%)	
Geographic region	Attica	0	97/160 (60.6%)	158/292 (54.1%)	0.024
	Thessaly		16/160 (10%)	57/292 (19.5%)	
	Crete		34/160 (21.3%)	45/292 (15.4%)	
	Macedonia		13/160 (8.1%)	32/292 (11%)	
Contact with other cats		57	131/141 (92.9%)	208/254 (81.8%)	0.003 ^a^
History of cat-fight trauma		253	15/55 (27.3%)	31/144 (21.5%)	0.39
Use of ectoparasiticides		187	56/91 (61.5%)	101/174 (58%)	0.583
Flea infestation		11	43/156 (27.6%)	44/285 (15.4%)	0.002 ^a^
Tick infestation		12	5/156 (3.2%)	2/284 (0.7%)	0.103 ^a^

^a^ Variables that were used in the logistic regression model (*p* value < 0.25).

**Table 3:** Multivariate analysis of signalment and historical data identified as factors associated with *Bartonella henselae* seropositivity and haemoplasma species PCR-positive status.

Variables	Categories	Positivity (%)	OR	CI	*p* Value
1. *Bartonella henselae*					
Age ^a^			1.1	1.03–1.18	0.005
Living conditions	Indoors	21/97 (21.6%)	Reference		
	Outdoors	129/333 (38.7%)	2.17	1.2–3.95	0.02
Geographic region	Thessaly	16/73 (21.9%)	Reference		
	Attica	97/255 (38%)	2.53	1.29–4.93	0.006
	Crete	34/79 (43%)	2.95	1.35–6.28	0.006
Flea infestation	No	113/354 (31.9%)	Reference		
	Yes	43/87 (49.4%)	1.73	1.02–2.92	0.041
2. Haemoplasma species					
Use of ectoparasiticides	No	21/80 (26.2%)	Reference		
	Yes	8/83 (9.6%)	0.29	0.12–0.72	0.007

^a^ In the present study, age was a continuous variable; OR: odds ratio; CI: confidence interval.

**Table 4:** Univariable associations between the infection status of 242 cats tested for haemoplasma species by PCR and the signalment and historical data that were collected using a standardised questionnaire (Questionnaire S1).

			Haemoplasma Species PCR Status		
Variables	Categories	Missing Data	Positive (%)	Negative (%)	*p* Value
Sex	Male	0	25/46 (54.3%)	105/196 (53.6%)	0.924
	Female		21/46 (45.7%)	91/196 (46.4%)	
Neutered		3	11/45 (24.4%)	60/194 (30.9%)	0.391
Breed	Purebred	6	0/44 (0%)	44/44 (100%)	1
	Crossbreed		2/190 (1%)	190/192 (99%)	
Age (years)		9	2 (0.13–8)	1 (0.13–15)	0.705
Cat acquisition	Client-owned		9/32 (28.1%)	47/152 (30.9%)	0.879
	Stray	58	20/32 (62.5%)	92/152 (60.5%)	
	Cattery		3/32 (9.4%)	11/152 (7.2%)	
	Pet shop		0/32 (0%)	2/152 (1.3%)	
Current ownership	Client-owned		22/45 (48.9%)	115/195 (59%)	0.453
	Stray	2	20/45 (44.4%)	71/195 (36.4%)	
	Cattery		3/45 (6.7%)	9/195 (4.6%)	
Living conditions	Indoors	7	2/44 (4.5%)	28/191 (14.7%)	0.07 ^a^
	Outdoors		42/44 (95.5%)	163/191 (85.3%)	
Living area	Urban	8	31/44 (70.5%)	145/190 (76.3%)	0.417
	Rural		13/44 (29.5%)	45/190 (23.7%)	
Geographic region	Attica		12/46 (26.1%)	82/196 (41.8%)	0.238
	Thessaly	0	13/46 (28.3%)	47/196 (24%)	
	Crete		16/46 (34.8%)	54/196 (27.6%)	
	Macedonia		5/46 (10.9%)	13/196 (6.6%)	
Contact with other cats		11	43/44 (97.7%)	174/187 (93%)	0.479
History of cat-fight trauma		120	4/19 (21.1%)	24/103 (23.3%)	1
Use of ectoparasiticides		79	8/29 (27.6%)	75/134 (56%)	0.006 ^a^
Flea infestation		6	14/45 (31.1%)	49/191 (25.7%)	0.457
Tick infestation		7	1/45 (2.2%)	4/190 (2.1%)	1

PCR: polymerase chain reaction. ^a^ Variables that were used in the logistic regression model (*p* value < 0.25).

## Supplementary Materials

The following supporting information can be downloaded at: https://www.mdpi.com/article/10.3390/vetsci9070337/s1, Questionnaire S1: Questionnaire for cat owners.
