# Supplementary material for: Prevalence and Risk Factors for Bartonella spp. and Haemoplasma Infections in Cats from Greece
Source: Vet Sci. 2022 Jul 3;9(7):337. doi: 10.3390/vetsci9070337 (PMC9315813; doi:10.3390/vetsci9070337)
Supplement: Supplementary file 1 [file vetsci-09-00337-s001.zip › Questionnaire S1.pdf]

ID:

Date:

QUESTIONNAIRE FOR CAT OWNER
(SIGNALMENT AND HISTORICAL DATA)

**Name of owner:**	**Name of cat:**
**Address:**	**Sex: Male intact € Male neutered €**<br>**Female intact € Female neutered €**
**Veterinarian:**	**Breed:**
**Vet clinic:**	**Age:**
**Telephone number :**	**Body weight (kg):**

1. **Living conditions**

   Indoors € Outdoors €

2. **Living area**

   Urban € Rural €

3. **Current ownership**

   Client-owned € Stray € Cattery €

4. **Travel history during the last year**

   Yes € No € I do not know €

   - If yes, where; …………………………………………….

5. **Cat acquisition**

   Stray € Non-stray €

6. **Contact with other cats**

   Yes € No € I do not know €

   - If yes, with how many?

7. **History of cat-fight trauma**

   Yes € No € I do not know €

8. **Vaccinations**

   Panleukopenia € Calicivirus € Herpesvirus € Chlamydia €

   Rabies € Bordetella € FIP € FeLV €

   - Fully vaccinated € Partially vaccinated € Unvaccinated € I do not know €

9. **Use of ectoparasiticides**

   Regular use € Intermittent use € Non-use € I do not know €

10. **Have you ever noticed flea infestation?**

    Yes € No € I do not know €

11. **Have you ever noticed tick infestation?**

Yes €　　No €　　I do not know €

12. Diet

Canned food €　　Dry food €　　Home cooked diet €　　Raw meat €

13. Appetite during the last three weeks

Increased €　　Decreased €　　Normal €　　I do not know €

14. Weight loss during the last three weeks

Yes €　　No €　　I do not know €

15. Is your cat currently healthy?

Yes €　　No €

PRIOR MEDICAL PROBLEMS

(Free text response)

CHIEF PRESENTING COMPLAINT

- Disease onset-acute or gradual
- progression and duration
- intervening signs
- present health status: weight loss or weight gain, attitude, activity level, appetite status, urination and defecation characteristics, amounts of water intake

PREVIOUS TREATMENTS

(Free text response)
